# Metabolic Profiling of Nine *Mentha* Species and Prediction of Their Antioxidant Properties Using Chemometrics

**DOI:** 10.3390/molecules24020258

**Published:** 2019-01-11

**Authors:** Yun Ji Park, Seung-A Baek, Yongsoo Choi, Jae Kwang Kim, Sang Un Park

**Affiliations:** 1 Department of Crop Science, Chungnam National University, 99 Daehak-ro, Yuseong-gu, Daejeon 34134, Korea; yunji0825@hanmail.net; 2 Division of Life Sciences and Convergence Research Center for Insect Vectors, Incheon National University, Incheon 22012, Korea; bsa1103@inu.ac.kr; 3 Systems Biotechnology Research Center, Korea Institute of Science and Technology (KIST), Gangneung 25451, Korea; yongsoo.choi@kist.re.kr

**Keywords:** *Mentha*, metabolite analysis, multivariate analysis, mint plant, antioxidant activity

## Abstract

*Mentha* species are well recognized for their medicinal and aromatic properties. The comprehensive metabolite profiles of nine *Mentha* species have been determined. The extracts of these *Mentha* species were also screened for antioxidant and free radical scavenging activities. Forty-seven hydrophilic and seventeen lipophilic compounds were identified and quantified from the selected *Mentha* species. Also, eleven phenolic compounds, riboflavin and eight carotenoids were present, and their composition and content varied among the various *Mentha* species. The different *Mentha* species exhibited a range of antioxidant potencies. Horse mint especially exhibited the strongest antioxidant capacities (1,1-diphenyl-2-picryl-hydrazyl (DPPH), hydrogen peroxide, and reducing power assay) among the nine *Mentha* species. A difference between different samples from the same species was not observed by multivariate analysis. A high correlation between metabolites involved in closely linked biosynthetic pathways has been indicated. The projection to latent structure method, using the partial least squares (PLS) method, was applied to predict antioxidant capacities based on the metabolite profiles of *Mentha* leaves. According to the PLS analysis, several carotenoid contents, such as *E*-β-carotene, 9*Z*-β-carotene, 13*Z*-β-carotene and lutein, as well as phenolic compounds, showed a positive relationship in reducing the power of *Mentha* extracts. Horse mint is a good candidate because of its high antioxidant efficacy among the nine *Mentha* species included in the study.

## 1. Introduction

The genus *Mentha* (Lamiaceae), which is commonly known as mint, has been recognized for its medicinal, therapeutic and aromatic properties since ancient times [1]. There are approximately 25–30 species of *Mentha* [2]. These plants are of great economic importance because the aerial parts of these plants are used in cooking and in the production of aromatic products, daily items and pharmaceuticals [3]. *Mentha* species have antidiarrheal, antimicrobial, antioxidant and anti-inflammatory properties, in addition to their therapeutic potential in the cardiovascular field of medicine [4].

Human cells are affected by the reactive oxygen species produced during metabolism under physiological conditions. Antioxidants neutralize the free radicals, which destroy lipids, proteins, and nucleic acids. Several human disorders such as atherosclerosis, arthritis, ischemia, gastritis, cancer and many tissue injuries, such as a central nervous system injury, result from the destructive action of free radicals [5]. Non-enzymatic compounds, including ascorbic acid, tocopherol and β-carotene, which inhibit the formation of free radicals, can also be used as antioxidants. Antioxidants, which possess the ability to prevent damage caused by free radical-induced oxidative stress, are critical for the survival of organisms [6].

Plant metabolites, including carbohydrates, organic and amino acids, vitamins, hormones, flavonoids, phenols and glucosinolates, are fundamental for plant development, stretch adjustment and protection. Apart from the importance of these compounds for the plant itself, they determine the nutrition value, color, taste, smell, antioxidative, anticarcinogenic, antihypertensive, calming, antimicrobial, immunostimulating and cholesterol lowering properties of the various plant parts. A considerable amount of research has already gone into the identification, biochemical characterization, localization and health benefits of plant metabolites [7]. Making use of the recent advances in metabolite profiling will increase our understanding of metabolic systems by recognizing the interrelationships between various metabolites [8]. In rice, it was studied using metabolic profiling that the flavonoids had a positive correlation with carotenoids [9]. Moreover, the carotenoids showed a positive correlation with glucosinolates in Chinese cabbage [10]. However, there are few studies on the relationship between metabolites in *Mentha* species.

**Figure 1:**
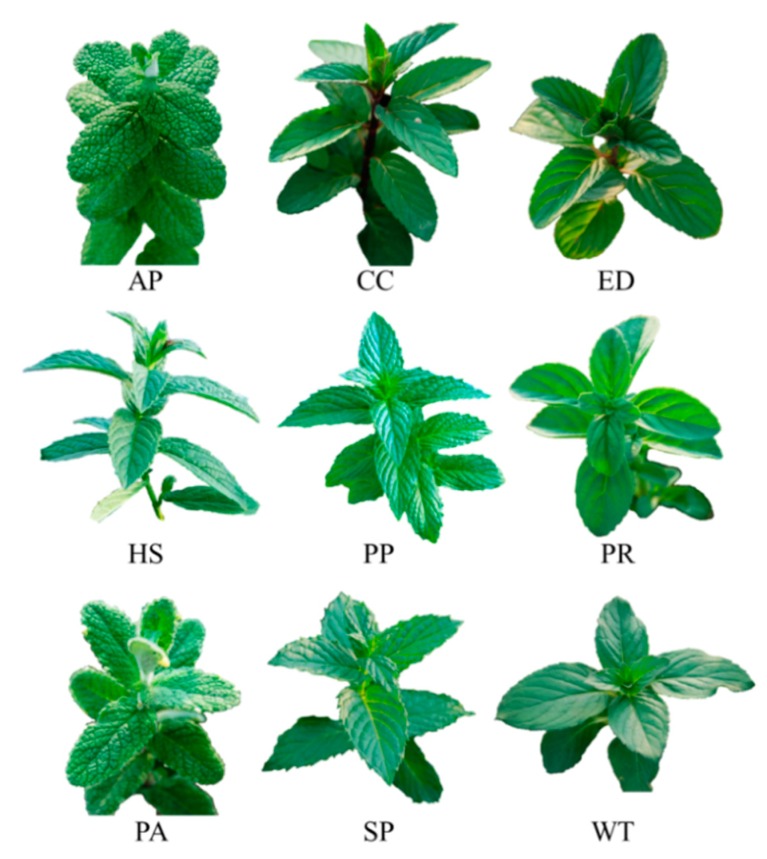
Nine *Mentha* species. AP, apple mint; CC, chocolate mint; ED, eau de cologne mint; HS, horse mint; PP, peppermint; PR, pennyroyal mint; PA, pineapple mint; SP, spearmint; WT, water mint.

In plants, the building blocks of secondary metabolites are derived from primary metabolism. The objective of the current study was to analyze 84 metabolites, including the primary and secondary metabolites, of nine *Mentha* species: Peppermint (*Mentha* × *piperita*), water mint (*M. aquatica*), apple mint (*M. suaveolens*), spearmint (*M. spicata*), chocolate mint (*M. piperita* ‘Chocolate’), pineapple mint (*M. suaveolens* ‘Variegata’), horse mint (*M. longifolia*), eau de cologne mint (*M. piperita* f. citrata) and pennyroyal mint *(M. pulegium*) (Figure 1). We have also assessed the antioxidant activity of the extracts of these *Mentha* species and examined the relationship between the metabolite composition and antioxidant activity using chemometrics.

## 2. Results

### 2.1. Metabolic Profiling of Nine Mentha spp. on the Basis of GC-MS Database

Hydrophilic metabolites in the nine *Mentha* spp. were identified by gas chromatography-mass spectrometry (GC-MS). Peaks were identified using an in-house library and quantified using the peak area ratios relative to the internal standard (IS). In total, 47 hydrophilic metabolites, including 18 amino acids, 18 organic acids, 7 sugars, 3 sugar alcohols and 1 amine were detected (Table S1 and Figure S1).

Our analysis detected 17 lipophilic metabolites, including 9 policosanols, 3 sterols, 3 tocopherols and 2 amyrins (Figures S2, S3 and Table S2). C30 (triacontanol, 12.04–90.54 μg/g dry weight (DW)), β-sitosterol (585.25–986.15 μg/g DW) and α-tocopherol (71.79–141.58 μg/g DW) were the predominant policosanol, sterol and tocopherol, respectively, in all samples. Water mint had the highest content of total policosanol (169.97 μg/g DW). The highest content of total sterol, tocopherol and amyrin was found in apple mint (1234.07 μg/g DW), horse mint (145.87 μg/g DW) and water mint (402.68 μg/g DW), respectively.

### 2.2. Secondary Metabolite Profiling in the Nine Mentha spp.

The composition and content of the phenolic compounds in the nine *Mentha* species was determined by high performance liquid chromatography (HPLC) analysis. In total, 11 phenolic compounds (including rosmarinic acid, chlorogenic acid, caffeic acid, epicatechin, *p*-coumaric acid, ferulic acid, benzoic acid, rutin, trans-cinnamic acid, quercetin and kaempferol) were detected (Table 1 and Figure S4). The amount of each constituent varied between the given *Mentha* species studied. Rosmarinic acid constituted the largest component among the 11 phenolic compounds. The concentration of rosmarinic acid was the highest in peppermint. The rosmarinic acid content was 16-fold higher in peppermint than that in pennyroyal mint, which contained the lowest level of the constituent. Most of the hydroxycinnamic acids, such as chlorogenic acid, caffeic acid and ferulic acid, exhibited similar patterns. However, the level of *p*-coumaric acid in pennyroyal mint was approximately 100 times higher than that in others. The highest concentration of benzoic acid was detected in pennyroyal mint. It was not detected in chocolate mint. The trans-cinnamic acid content was significant in all the species studied. The highest amounts of trans-cinnamic acid were found in pineapple mint, peppermint and spearmint. The horse mint predominantly contained rutin. Chocolate mint, spearmint, apple mint and water mint also had large amounts of rutin. Among the flavonols, quercetin was predominant in apple mint and peppermint, whereas pennyroyal mint and spearmint contained high levels of kaempferol. Epicatechin, a flavan-3-ol, was present at similar levels.

From the time of their discovery, phenolic compounds have been a distinguishable trait of plants. These compounds are crucial for the organoleptic and nutritive quality of fruits, including the color, taste, aroma, flavor and astringency [11]. Phenolic compounds have been demonstrated to vary between cultivars and due to other factors, including the maturity stage and amount of light exposure. The profiling of phenolic compounds has been a useful parameter to discriminate fruit parts [12]. Interest in polar compounds such as phenolic acids and flavonoids has spiked recently because of their stability during boiling and storage. Many studies have already isolated and identified a wide range of phenolic compounds from different *Mentha* species, including *M. spicata*, *M. aquatica*, *M. citrate* and others [13]. For instance, the genus *Mentha* contains caffeic acid derivatives, flavones, flavanones and their glycosidic forms. Accordingly, rosmarinic acid was indicated as the major component [3]. These findings are consistent with the results of the present study. Various polypheonols were identified and quantified in the *M. pulegium* extracts. However, the content of rosmarinic acid (0.287 mg/g) was lower than in our findings [14]. Chemical profiling of the *Mentha* species has also been used as an additional marker for confirming the parental origin of hybrids of naturally occurring and cultivated species [15].

Riboflavin was quantitated by HPLC analysis (Figure S5). Table 2 shows the riboflavin content in the nine different *Mentha* species. The highest concentration, 25.40 μg/g DW, was found in spearmint. Pineapple mint and apple mint also contained high levels of riboflavin. Peppermint contained the lowest amount of riboflavin at 15.96 μg/g DW. The difference in riboflavin content between spearmint and peppermint was indicated as a 1.59-fold change.

Four types of carotenes (α-carotene, *E*-β-carotene, 9*Z*-β-carotene and 13*Z*-β-carotene) and four types of xanthophylls (antheraxanthin, lutein, violaxanthin and zeaxanthin) were isolated and quantified by HPLC analysis from the *Mentha* species included in the study (Table 2 and Figure S6). Of these, lutein and E-β-carotene were the major carotenoids that were present in large quantities. The pattern of individual carotenes was similar in all species. Eau de cologne mint, pennyroyal mint, chocolate mint and spearmint all contained large amounts of carotenes, whereas apple mint, pineapple mint and water mint contained lower quantities of carotenes. The levels of total xanthophylls and the amounts of each of the individual constituents varied among the different *Mentha* species. Spearmint had higher levels of antheraxanthin than that of other species. The highest zeaxanthin concentration was found in spearmint, whereas the lowest level was observed in pennyroyal mint. In contrast, pennyroyal mint showed the highest concentration of violaxanthin, followed by spearmint, apple mint, chocolate mint, horse mint, pineapple mint, peppermint, eau de cologne mint and water mint. The levels of lutein were similar in all the nine *Mentha* species.

Carotenoids are natural pigments, usually of yellow, orange or red color, and play crucial roles in photosynthesis, photoprotection, development, as stress hormones and as signaling molecules in plants [16,17]. Moreover, certain carotenoids are known as precursors of vitamin A, great antioxidants and to be essential for human health [17]. Straumite et al. (2015) [18] have reported that the highest contents of carotenoids in *M. spicata* were observed in the leaf and stem. In addition, several *Mentha* varieties, including *M. suaveolens*, *M. suaveolens* ‘Variegata’ and *M. piperita* ‘Bavarian’) have shown significantly higher concentrations of carotenoids [18].

### 2.3. In Vitro Antioxidant Assays

**Figure 2:**
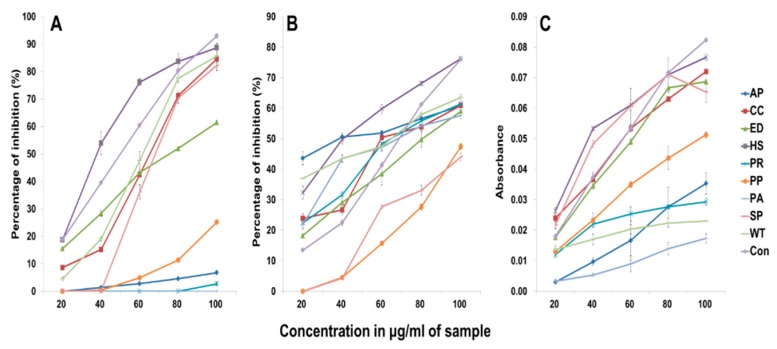
Antioxidant activity of different *Mentha* extracts. Ascorbic acid was used as the control. (**A**) 1,1-diphenyl-2-picryl-hydrazyl (DPPH) assay; (**B**) hydrogen peroxide assay; (**C**) reducing power assay. AP, apple mint; CC, chocolate mint; ED, eau de cologne mint; HS, horse mint; PP, peppermint; PR, pennyroyal mint; PA, pineapple mint; SP, spearmint; WT, water mint.

1,1-Diphenyl-2-picryl-hydrazyl (DPPH) radical scavenging activity is one of the most efficient methods for screening the antioxidant activity of plant extracts. The results of the analysis of the DPPH free radical scavenging activity in the various *Mentha* crude extracts are shown Figure 2A. The activity of these extracts was concentration dependent and was comparable to that of the ascorbic acid standard. Horse mint extract, at a concentration of 100 μL/mL, showed an 88.6% activity compared to the 93.03% activity of the ascorbic acid standard at a concentration of 100 μL/mL. Horse mint was followed by water mint, chocolate mint, spearmint, eau de cologne mint, peppermint and apple mint. Pennyroyal mint and pineapple mint had no antioxidant activity.

Additionally, the results for hydrogen peroxide radical scavenging activity are depicted in Figure 2B. Hydrogen peroxide can easily cross cell membranes. Once inside the cell, H_2_O_2_ can react with Fe^2+^ and possibly Cu^2+^ ions to form hydroxyl radicals, which may be the reason for many of its toxic effects. The hydrogen peroxide radical scavenging activity was concentration dependent. It was highest (76.2%) in a 100 μL/mL concentration of horse mint crude ethanol extract, compared to the 76.1% of 100 μL/mL ascorbic acid. Horse mint was followed by water mint, pennyroyal mint, chocolate mint, apple mint, eau de cologne mint, pineapple mint and peppermint. Spearmint exhibited the lowest hydrogen peroxide radical scavenging activity compared to that of the other species.

The reducing power of the nine *Mentha* species was compared to that of ascorbic acid (Figure 2C). All the extracts showed good reducing power, as indicated by the absorbance measurements of the reaction mixtures at the end of the incubation period. The presence of reducing agents causes the conversion of the Fe^3+^/ferricyanide complex used in this method to the ferrous form. The reducing capacity of an extract may serve as a relevant indicator of its antioxidant potential. Among the *Mentha* species studied, horse mint displayed higher reducing activity, followed by chocolate mint, eau de cologne mint, spearmint, peppermint, apple mint, pennyroyal mint, water mint and pineapple mint.

An earlier study has shown that extracts of different *Mentha* species are good sources of natural antioxidants but vary in the degree of their antioxidant potential [3]. It has been shown that water extract of *M. pulegium* is comparable to the synthetic antioxidant, butylated hydroxytoluene (BHT), and can be considered as a suitable alternative for BHT [19]. Nickavar et al. (2010) reported that the ethanol extract of *M. piperita* has the strongest DPPH scavenging activity among five *Mentha* species (*M. piperita*, *M. pulegium*, *M. rotundifolia*, *M. spicata* and *M. longifolia*) [20]. This result is in contrast to our findings. According to our study, the highest DPPH scavenging activity was detected in horse mint, otherwise known as *M. longifolia*. Many studies have reported the chemical composition of plant extracts and their antioxidant activity. However, it is very complicated to interpret the data because of differences in methods which are based on different mechanisms used for the evaluation of the antioxidant activity. The results of the antioxidant activity analysis of the same plant can vary significantly depending on the method used [21]. Therefore, it is important to evaluate and compare the antioxidant activities of the different species, measured using similar approaches.

### 2.4. PCA, HCA, and PLS

**Figure 3:**
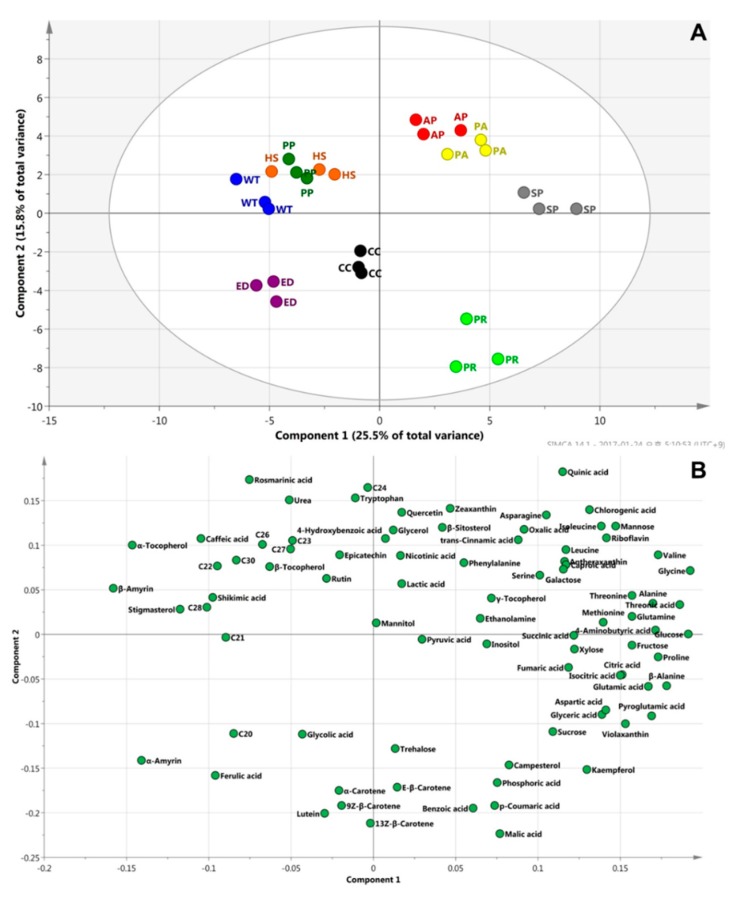
Principal component analysis (PCA) analysis of the metabolite profiles. (**A**) Score plot; (**B**) loading plot. AP, apple mint; CC, chocolate mint; ED, eau de cologne mint; HS, horse mint; PP, peppermint; PR, pennyroyal mint; PA, pineapple mint; SP, spearmint; WT, water mint; C20, eicosanol; C21, heneicosanol; C22, docosanol; C23, tricosanol; C24, tetracosanol; C26, hexacosanol; C27, heptacosanol; C28, octacosanol; C30, triacontanol.

Principal component analysis (PCA) was used to confirm the differences between samples and the contribution of the metabolites to clustering. Figure 3 shows that there was no difference between different samples from the same species. A large separation among samples indicates a distinct difference. According to the loading plot, the metabolites responsible for the highest contribution to the cluster of three species (horse mint, peppermint and water mint) were policosanols. Benzoic acid, *p*-coumaric acid and malic acid contributed to the separation of pennyroyal mint from the other samples. This means that pennyroyal mint has a lot of benzoic acid, *p*-coumaric acid and malic acid, compared to that of the other samples.

**Figure 4:**
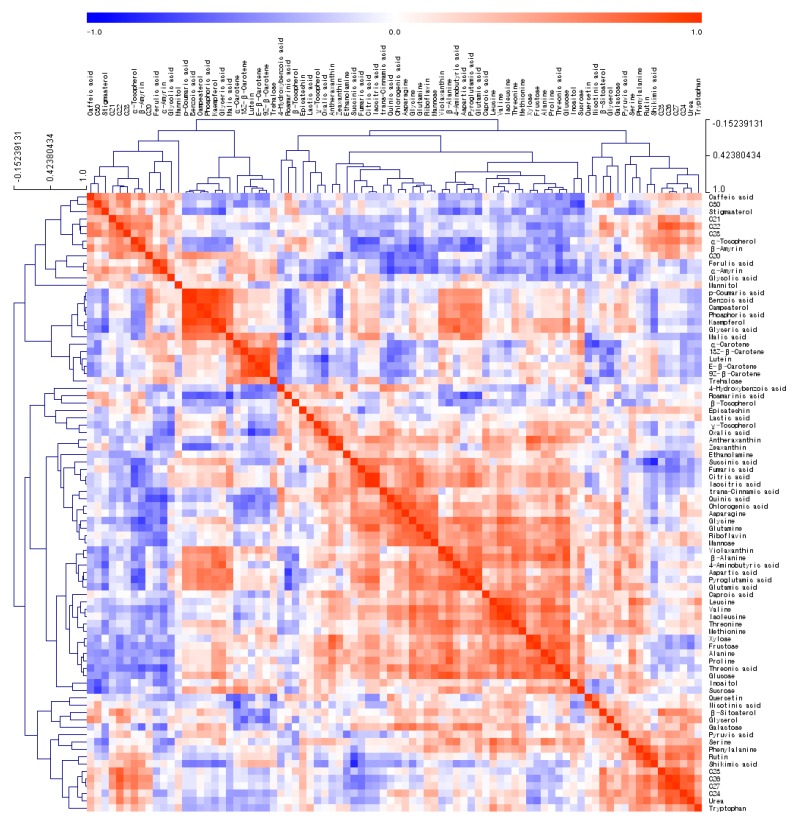
Hierarchical clustering analysis (HCA) of compound data from the nine *Mentha* spp. Each square indicates the Pearson’s correlation coefficient of a pair of compounds and the value of the correlation coefficient is represented by the intensity of blue or red colors, as indicated on the color scale. C20, eicosanol; C21, heneicosanol; C22, docosanol; C23, tricosanol; C24, tetracosanol; C26, hexacosanol; C27, heptacosanol; C28, octacosanol; C30, triacontanol.

Hierarchical clustering analysis (HCA) was performed using Pearson’s correlation and average linkage to examine relationships between the concentrations of the 84 metabolites. In HCA, when metabolites are located close to each other, this indicates a high correlation (Figure 4). For example, isocitric acid, an intermediate in the citric acid cycle, was located next to citric acid (r = 0.9967; *p* < 0.0001). Benzoic acid and *p*-coumaric acid (r = 0.9897; *p* < 0.0001) are related to salt tolerance in plants [22]. The carotenoids were also clustered in HCA.

**Figure 5:**
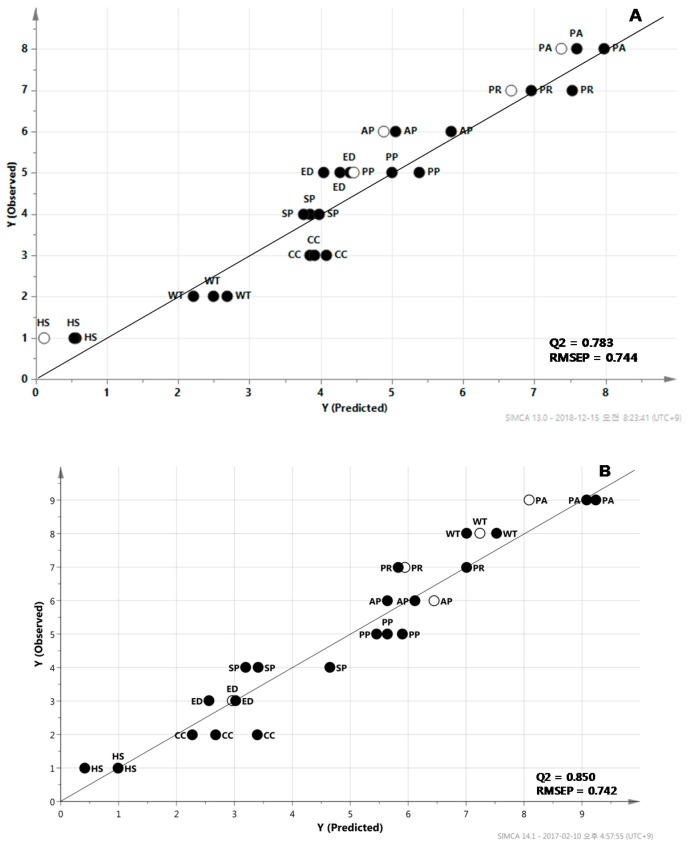
The partial least squares (PLS) predictive models constructed from the nine *Mentha* spp. as a training set (filled circle) for predicting the ranking of DPPH free radical scavenging activity (**A**) and reducing power (**B**) based on metabolite profiles from the *Mentha* samples. A predicted result after five samples of test sets (open circle) was projected on to the model. AP, apple mint; CC, chocolate mint; ED, eau de cologne mint; HS, horse mint; PP, peppermint; PR, pennyroyal mint; PA, pineapple mint; SP, spearmint; WT, water mint.

**Figure 6:**
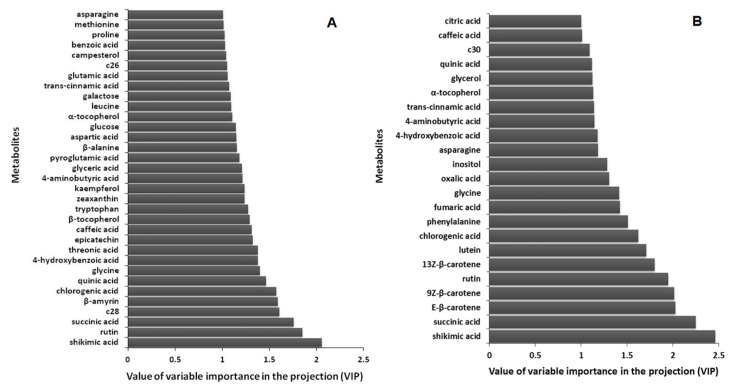
The influence of variables used to create DPPH free radical scavenging (**A**) and the reducing power (**B**) predictor for *Mentha* spp. C26, hexacosanol; C28, octacosanol; C30, triacontanol.

In the partial least squares (PLS) method, the ranking of antioxidant capacity (including DPPH free radical scavenging activity and reducing power) and the metabolite quantities were used as the dependent (*y*) and independent (*x*) variables, respectively. The 84 metabolites from the nine *Mentha* spp. were divided into 22 training set samples and 5 test set samples (Figure 5). In the PLS prediction model, a cross-validated correlation coefficient (Q2) > 0.5 indicates a good model [23]. In these prediction models, Q2 was 0.78 and the root mean squared error of prediction (RMSEP) was 0.74. The variables important in the projection (VIP) indicate the contribution of each variable to the projection. Metabolites with a VIP value greater than 1 had a strong impact on the prediction model [24]. Shikimic acid was indicated to be the most important contributor to predicting the ranking of DPPH free radical scavenging activity and the reducing power of the *Mentha* species. Additionally, carotenoids (*E*-β-carotene, 9*Z*-β-carotene, 13*Z*-β-carotene and lutein) as well as phenylpropanoids (rutin and chlorogenic acid) were important to predicting the reducing power of the samples (Figure 6).

## 3. Discussion

Secondary metabolites such as phenolic compounds, ascorbic acid and carotenoids have an effect on the antioxidant properties of a medicinal plant [25]. For instance, a high correlation is observed between the DPPH scavenging activity and the total phenolic content (R2 > 0.989) [19]. Phenolic compounds act as scavengers of chain-breaking peroxyl-radicals, thus inhibiting lipid peroxidation [26]. Metabolomics can assist in dissecting the mechanism that regulates the conversion of primary metabolites into secondary metabolites in plants. In our results, shikimic acid and phenylalanine, precursors used in the phenylpropanoid biosynthetic pathway, were important for creating the prediction models of antioxidant capacities (Figure 5 and Figure 6). Shikimic acid and phenylalanine were shown to have a high degree of correlation each other (r = 0.5935; *p* < 0.001). Rutin was also shown to have a high correlation with shikimic acid and phenylalanine (r = 0.8460; *p* < 0.0001 and r = 0.8417; *p* < 0.0001), respectively. Riboflavin can be used as a natural antioxidant to protect the human body against oxidative stress, especially from lipid peroxidation [27]. The antioxidant capacity of food carotenoids is involved in quenching singlet oxygen and free radical scavenging [28]. The results of our present study indicate a positive correlation between the total carotenoid content and the reducing power of *Mentha* extracts (Figure S7). The correlation coefficient (R2) was determined to be 0.7913. These results suggest that 79% of reducing power is due to the contribution of carotenoid compounds. This relationship was also confirmed by the PLS prediction model and VIP values (Figure 4 and Figure 5). However, there was no apparent association between the antioxidant potential and other secondary metabolites, such as riboflavin. Variability in the biological effectiveness is related to the chemical composition, genotype and cultivar of *Mentha* [13]. Even though numerous studies have already shown the potential of plant extracts as natural antioxidants, it has been difficult to interpret the relationship between the components and their antioxidant potential because of their chemical complexity [21].

There has been considerable interest in recent times in the role of plant-derived antioxidants in human health. Because of increasing consumer demand for natural and organic products rather than synthetic antioxidants, many research groups have focused on edible medicinal plants as natural sources of harmless and effective antioxidants in the food industry. There have been reports on the protective effect of natural antioxidants against oxidative stress [4]. Slowing down of the progression of chronic diseases in humans can be correlated to the intake of natural antioxidants [29]. The significant free radical scavenging capacity of crude extracts of aromatic plants has been well documented [21]. For instance, it has been determined that mint, beet and ginger have excellent antioxidant capabilities, comparable to that of synthetic agents [4]. It is known that a relationship between phenylpropanoids and antioxidant potential exists. Our results have provided the possibility of using a PLS prediction model for selecting plants with high antioxidant potential using metabolic profiling.

## 4. Materials and Methods

### 4.1. Plant Materials

Young seedlings of the nine *Mentha* species were obtained from the Seed Mall Co. (Seoul, Korea). The *Mentha* plants were grown in a greenhouse at the experimental farm of Chungnam National University (Daejeon, Korea). The plants were exposed to outdoor conditions during a period spanning March 2016 to June 2016. During cultivation, the average temperature was 16.45 °C, the relative humidity was 63.5% and the average precipitation was 85.7 mm, according to the data obtained from the Korea Meteorological Administration (http://web.kma.go.kr). The temperature and illumination were not controlled any further. Aerial parts of the plants that included leaves and stems were harvested and all samples were collected as three biological replicates. The samples were freeze-dried at −80 °C for 3 days (Ilshin Lab Co., Ltd., Dongducheon, Korea) and the dried plants were powdered finely using mortars and pestles.

### 4.2. Analysis of Hydrophilic and Lipophilic Metabolites using GC-MS

Hydrophilic metabolites were extracted by the method described by Kim et al. (2017) [30]. Ribitol was used as an internal standard (IS). The derivatization of hydrophilic compounds was carried out with methoxyamine hydrochloride and *N*-methyl-*N*-trimethylsilyl trifluoroacetamide (MSTFA). The GCMS-QP2010 Ultra system (Shimadzu, Kyoto, Japan) was used with a DB-5 column (30 m length, 0.25 mm inner diameter and 1 μm film thickness; Agilent, CA, USA). The split ratio was set to 1:10 and the flow rate of helium as the carrier gas was 1.1 mL/min. The injection temperature was 280 °C. The column temperature was held at 100 °C for 4 min and then raised to 320 °C at the rate of 10 °C/min, then held for 11 min at 320 °C. The ion source and interface temperatures were 200 °C and 280 °C, respectively. The scanned mass range was 45–600 *m*/*z*. Peak identification and analysis was performed using the LabSolutions GCMS solution software, version 4.11 (Shimadzu). A series of linear alkanes ranging from C6 to C26 were analyzed for retention indices (RI). To compare the retention time (RT), RI and mass spectra with analytes in the samples, the respective standards and Wiley9, NIST11 and OA TMS DB5 (Shimadzu) libraries were used (Table S1). Quantification was based on the peak area ratios relative to the IS peak area.

The extraction and GC-MS analysis of lipophilic metabolites was performed according to the method described by Kim et al. (2015) [31] with slight modifications. 5α-Cholestane was used as the IS. The derivatization of lipophilic compounds was also carried out with MSTFA. The GCMS-QP2010 Ultra system with a Rtx-5MS column (30 m length, 0.25 mm inner diameter and 0.25 μm film thickness; Agilent, CA, USA) was used to separate the lipophilic compounds. Helium was used as the carrier gas at a flow rate of 1 mL/min. The split ratio was set to 1:10. The injection temperature was 290 °C and the column temperature was 150 °C. The oven temperature program consisted of a hold period of 2 min at 150 °C, increasing at the rate of 15 °C/min to 320 °C and a final hold period of 10 min at 320 °C. The ion source and interface temperatures were 230 °C and 280 °C, respectively. The scanned mass range was 85–600 *m*/*z*. Peak identification was performed by comparing with RT and mass spectra of standards. Standards of policosanols were obtained from Sigma (St. Louis, MO, USA). Amyrins, phytosterols and tocopherols were obtained from Merck (Darmstadt, Germany). Standard calibration curves were used for quantification. Quantification was performed using selected ions, as described in Figure S3. The calibration curve range of standard was from 0.25 to 5.0 μg.

### 4.3. HPLC Analysis

#### 4.3.1. Phenolic Compounds

Phenolic compound standards were purchased from Sigma-Aldrich (St. Louis, MO, USA). Twenty milligrams of dried sample was extracted with 3 mL of 80% methanol (*v*/*v*). The crude mixture was ultrasonicated for 1 h at room temperature and then centrifuged at 12,000 rpm for 10 min. The final extract was filtered using a 0.45 μm Acrodisc syringe filter (Pall Corp.; Port Washington, NY, USA). The flow rate was maintained at 1 mL/min and a 280-nm wavelength was used for detection. Elution was performed using a binary gradient of mobile phase A (0.15% of acetic acid in H_2_O, *v*/*v*) and mobile phase B (methanol). The volume of the sample injected was 20 μL and column temperature was maintained at 30 °C for detection. The individual compounds were separated and identified in the Futecs model NS-4000 HPLC apparatus (Daejeon, Korea).

#### 4.3.2. Riboflavin

Standard stock solutions of riboflavin (Sigma, St. Louis, MO, USA) were prepared in 0.01 M HCl. Quantification was performed using calibration curves ranging from 0.01 to 1.00 μg/mL. Riboflavin was extracted from 10 mg of the freeze-dried sample suspended in 1 mL of H_2_O. After incubating at 78 °C for 20 min, the sample was placed on ice for 3 min and then centrifuged at 15,000 rpm and 4 °C for 10 min. The supernatant was filtered using a 0.2 μm filter (Advantec, Tokyo, Japan) and 20 μL of the filtrate was injected into a Waters e2695 separation module system (Waters Corporation, Milford, MA, USA) equipped with a Waters 2475 fluorescence detector (Waters Corporation, Milford, MA, USA) and a C18 column (250 mm length, 4.6 mm inner diameter, 5 μm pore size; Waters Corporation, Milford, MA, USA). Elution was performed using a binary gradient of mobile phase A (0.1% formic acid in H_2_O) and mobile phase B (0.1% formic acid in acetonitrile). The flow rate was 1 mL/min and the column temperature was 40 °C. Peaks were identified and quantified using the Empower 3 software (Waters Corporation).

#### 4.3.3. Carotenoids

Carotenoids were extracted and analyzed as described by Park et al. (2014) with several modifications [32]. An HPLC series 1100 system (Agilent, Germany) with a YMC Carotenoid S-3μm column (250 mm length, 4.6 mm inner diameter; YMC separation technology, Kyoto, Japan) was used for separation. The injection volume was 20 μL and flow rate was maintained at 1 mL/min for detection. The column temperature was set to 40 °C. Data were analyzed using ChemStation for LC 3D software, Rev. A. 10.02 (Agilent Technologies). Peak identification was performed by standard solutions. Quantification was performed using calibration curves ranging from 0.16 to 5.00 μg/mL. The carotenoid standards were obtained from Extrasynthese (Genay, France).

### 4.4. Antioxidant Activity

#### 4.4.1. Extraction

Ten grams of the powdered sample was soaked in 50 mL of ethanol for one day and the extracts were filtered using filter paper. The filtrates were evaporated using a rotary vacuum evaporator and the dried samples were stored at 4 °C for subsequent experiments.

#### 4.4.2. DPPH Assay

We prepared 0.15% DPPH in ice cold methanol. The reaction mixture contained 1.6 mL of methanol and various amounts of extracts (20, 40, 60, 80 and 100 μL) which were added to a 200 μL DPPH solution. The reaction mixture was incubated at 25 °C for 30 min in dark. The absorbance was then measured at 517 nm. Vitamin C was used as the standard. The DPPH radical scavenging activity was determined using the following formula. DPPH radical scavenging activity (%) = [(A_0_ − A_1_/A_0_) × 100], where A_0_ is the absorbance of the control and A1 is the absorbance of the sample.

#### 4.4.3. Hydrogen Peroxide Scavenging Activity

A solution of hydrogen peroxide (40 mM) was prepared in phosphate buffer (pH 7.4). The reaction mixture contained different amounts of extracts (20, 40, 60, 80 and 100 μL) in 1 mL of distilled water. The reaction mixtures were incubated at 25 °C for 10 min after the addition of 0.6 mL of H_2_O_2_. The absorbance was then measured at 560 nm. Ascorbic acid was used as the standard. The hydrogen peroxide scavenging activity of the extracts was calculated using the following formula. Hydrogen peroxide radical scavenging activity (%) = [(A_0_ − A_1_/A_0_) × 100], where A_0_ is the absorbance of the control and A_1_ is the absorbance of the sample.

#### 4.4.4. Reducing Power Analysis

Different volumes of the extracts (20, 40, 60, 80 and 100 μL) were mixed with a 2.5 mL 0.2 M phosphate buffer (pH = 6.6). Subsequently, 2.5 mL of 1% K_3_Fe(CN)_6_ was added and the mixture was incubated at 50 °C for 20 min. Next, 2.5 mL of 10% trichloroacetic acid was added to the mixture which was then centrifuged at 3000 rpm for 10 min. The supernatant (2.5 mL) was mixed with an equal volume of distilled water, to which 0.5 mL of 1% FeCl_2_ was added and the absorbance was measured at 700 nm. The optical density values increased with increasing sample concentrations, indicating an increase in the reducing power. Vitamin C was used as the standard. A standard vitamin C solution of 1 mg/mL concentration was prepared as the stock solution for all experiments.

### 4.5. Statistical Analysis

The content of hydrophilic compounds, lipophilic compounds, phenolic compounds, riboflavin and carotenoids in the nine *Mentha* species were determined by PCA using the SIMCA software (version 14.1; MKS Umetrics AB, Umeå, Sweden). PCA is a clustering method that assesses the relationships among the samples. The score plot shows reciprocal contrast and the loading plot explains the cluster separation. Using the SAS software package (version 9.4; SAS Institute, Cary, NC, USA), Pearson’s correlation coefficients were calculated and HCA was performed using the software Multi-Experiment Viewer version 4.9.0 (http://www.tm4.org/mev/). Projection to latent structure using PLS was performed using the SIMCA software. Quantitative data was used as independent variable (*x*) and ranking of DPPH scavenging activity and reducing power were used as dependent variable (*y*). All experiments were carried out for three biological replicates. Each result is presented as the mean ± standard deviation.

## 5. Conclusions

To summarize, we have determined the primary and secondary metabolite profiles, including those of phenolic compounds, riboflavin, and carotenoids to determine the diversity among the phytochemicals and to analyze the relationships among their contents. Additionally, we screened the antioxidant and free radical scavenging properties of nine *Mentha* species. Cirlini et al. (2016) have reported the phenolic and volatile composition of a spearmint extract developed utilizing selective breeding to yield high amounts of rosmarinic acid [33]. However, few studies have reported on the variation in the characterization of carotenoids in different *Mentha* species [18]. Eleven phenolic compounds and eight carotenoids were identified and quantified through HPLC analysis. Forty-seven hydrophilic and seventeen lipophilic metabolites were identified using GC-MS. The composition of the various constituents and their antioxidant potential varied among the extracts of the different *Mentha* species. We developed good prediction models of antioxidant capacity from metabolic profiling using PLS, indicating that metabolomics should be a useful tool to predict plant quality. Our results suggest that *Mentha* species can be used as natural food preservatives, pharmaceuticals and cosmetic industry resources because of their strong antioxidant potential. Horse mint (*M. longifolia*) especially displayed the highest antioxidant efficacy, appearing to be a great variety amongst the nine *Mentha* species studied. Further research is required to establish a safe and effective method of mass producing antioxidant-rich *Mentha* species, which are of value to various industries.

## Figures and Tables

**Table 1:** Composition and content of phenolic compounds in different *Mentha* species (μg/g DW).

*Mentha* spp.	Chlorogenic Acid ^1^	Caffeic Acid	Epicatechin	*p*-Coumaric Acid	Ferulic Acid	Benzoic Acid	Rutin	*trans*-Cinnamic Acid	Quercetin	Kaempferol	Rosmarinic Acid
AP	290.70 ± 64.18	51.65 ± 12.24	131.35 ± 56.70	4.40 ± 0.38	29.55 ± 1.82	109.25 ± 86.71	1920.90 ± 45.16	8.25 ± 0.65	9485.89 ± 224.06	160.35 ± 24.10	27.73 ± 5.61
CC	168.50 ± 1.96	35.45 ± 2.68	101.70 ± 11.24	6.80 ± 2.05	42.71 ± 7.76	n.d. ^2^	3470.10 ± 165.59	46.55 ± 6.18	397.10 ± 48.51	14.50 ± 5.38	19.85 ± 3.53
ED	168.95 ± 0.77	40.85 ± 5.95	86.85 ± 3.13	4.65 ± 0.45	77.65 ± 9.41	210.10 ± 46.39	140.65 ± 17.21	14.66 ± 0.71	558.56 ± 134.59	9.50 ± 0.09	14.94 ± 0.58
HS	170.90 ± 7.75	58.50 ± 13.39	224.15 ± 44.48	6.15 ± 0.98	38.70 ± 3.74	25.84 ± 3.15	11,659.20 ± 408.36	6.50 ± 2.26	347.25 ± 139.14	14.35 ± 0.62	18.68 ± 2.15
PP	174.38 ± 6.22	42.10 ± 15.22	232.3 ± 39.64	3.95 ± 1.39	25.90 ± 2.61	8.40 ± 3.00	57.75 ± 14.92	207.20 ± 74.86	5092.24 ± 269.96	65.40 ± 9.83	42.44 ± 3.80
PR	190.15 ± 6.98	30.60 ± 1.33	108.40 ± 4.31	396.95 ± 82.69	61.20 ± 7.30	2277.19 ± 107.36	70.05 ± 21.27	4.25 ± 0.88	121.54 ± 29.74	749.80 ± 112.07	2.53 ± 0.18
PA	352.30 ± 11.78	45.65 ± 3.32	95.90 ± 11.78	7.00 ± 1.11	26.05 ± 4.07	28.09 ± 3.86	37.65 ± 5.40	441.15 ± 51.90	177.60 ± 37.87	104.05 ± 9.61	18.89 ± 0.81
SP	238.90 ± 7.73	40.90 ± 6.81	177.70 ± 2.93	14.55 ± 0.98	27.45 ± 3.13	0.79 ± 0.26	3126.90 ± 127.85	196.80 ± 11.86	1071.10 ± 121.53	259.40 ± 16.29	26.47 ± 2.99
WT	177.60 ± 11.44	94.55 ± 4.58	103.70 ± 16.63	7.30 ± 2.34	77.90 ± 14.56	152.93 ± 62.03	1059.95 ± 189.02	8.55 ± 2.85	18.00 ± 6.00	10.75 ± 1.50	37.74 ± 2.08

^1^ The data are presented in this table as mean ± standard deviation of three biological replicates (*n* = 3). ^2^ n.d., not detectable. AP, apple mint; CC, chocolate mint; ED, eau de cologne mint; HS, horse mint; PP, peppermint; PR, pennyroyal mint; PA, pineapple mint; SP, spearmint; WT, water mint.

**Table 2:** Composition and content of carotenoids and riboflavin in different *Mentha* species (μg/g DW).

*Mentha* spp.	Riboflavin	Carotenoids							
		Violaxanthin ^1^	Antheraxanthin	Lutein	Zeaxanthin	13*Z*-β-Carotene	α-Carotene	*E*-β-Carotene	9*Z*-β-Carotene
AP	23.48 ± 0.04 ^1^	19.64 ± 0.17	3.90 ± 0.04	248.49 ± 0.10	13.25 ± 0.09	40.95 ± 1.88	2.94 ± 0.14	221.65 ± 7.77	33.92 ± 0.73
CC	20.39 ± 0.47	19.13 ± 0.21	4.23 ± 0.04	314.69 ± 6.84	13.79 ± 0.24	56.17 ± 0.46	3.94 ± 0.04	272.28 ± 15.16	41.80 ± 1.53
ED	17.67 ± 1.54	16.42 ± 1.03	3.23 ± 0.22	306.35 ± 15.61	11.63 ± 0.22	62.57 ± 3.64	4.64 ± 0.28	267.33 ± 7.50	42.62 ± 1.63
HS	21.65 ± 2.02	17.61 ± 0.22	4.06 ± 0.09	277.40 ± 3.87	12.57 ± 0.07	54.93 ± 0.24	4.43 ± 0.07	222.53 ± 1.70	37.24 ± 0.95
PP	15.96 ± 1.14	16.48 ± 0.41	5.78 ± 0.17	241.22 ± 10.60	15.12 ± 0.50	47.30 ± 2.05	4.30 ± 0.12	185.83 ± 7.59	34.80 ± 1.60
PR	19.20 ± 0.38	21.28 ± 1.30	3.60 ± 0.29	283.43 ± 11.25	9.15 ± 0.13	59.98 ± 2.21	4.68 ± 0.54	239.28 ± 11.99	38.52 ± 3.15
PA	25.33 ± 0.87	16.51 ± 0.32	5.92 ± 0.11	248.98 ± 4.08	14.00 ± 0.23	40.38 ± 2.13	3.74 ± 0.11	165.30 ± 2.00	31.03 ± 0.34
SP	25.40 ± 2.13	20.77 ± 0.45	10.14 ± 0.16	269.17 ± 5.42	19.17 ± 0.30	57.43 ± 3.89	4.53 ± 0.22	236.92 ± 9.81	37.64 ± 2.15
WT	18.25 ± 4.18	15.88 ± 0.16	4.94 ± 0.08	257.31 ± 9.84	15.20 ± 0.32	42.51 ± 0.74	3.97 ± 0.06	171.88 ± 9.86	32.12 ± 1.93

^1^ The data are presented in this table as mean ± standard deviation of three biological replicates (*n* = 3). AP, apple mint; CC, chocolate mint; ED, eau de cologne mint; HS, horse mint; PP, peppermint; PR, pennyroyal mint; PA, pineapple mint; SP, spearmint; WT, water mint.

## Supplementary Materials

The following are available online.
