# Supplementary material for: Metabolic Profiling of Nine Mentha Species and Prediction of Their Antioxidant Properties Using Chemometrics
Source: Molecules. 2019 Jan 11;24(2):258. doi: 10.3390/molecules24020258 (PMC6359624; doi:10.3390/molecules24020258)

Table 1. Gas chromatographic and mass spectrometric data of the identified hydrophilic compounds.

Compound	RT[a]	RRT[b]	RI[c]	Quantification ion[d]	Other characteristic ions
Amino acids					
Alanine	9.109	0.499	1106	190	218, 147, 116
Valine	11.133	0.610	1222	218	156, 144, 133
Leucine	12.033	0.659	1278	158	232, 218, 102
Isoleucine	12.401	0.679	1301	158	232, 218, 147
Proline	12.475	0.683	1306	216	244, 147, 142
Glycine	12.623	0.691	1316	248	276, 174, 147
Serine	13.376	0.733	1367	306	278, 218, 204
Threonine	13.804	0.756	1396	291	218, 147, 117
β-Alanine	14.355	0.786	1436	174	290, 248, 147
Aspartic acid	15.594	0.854	1529	334	306, 232, 188
Methionine	15.606	0.855	1530	293	250, 176, 128
4-Aminobutyric acid	15.746	0.863	1541	304	216, 174, 147
Pyroglutamic acid	15.703	0.860	0	156	258, 230, 147
Glutamic acid	16.83	0.922	1628	363	348, 246, 128
Phenylalanine	16.997	0.931	1642	218	266, 192, 141
Asparagine	17.474	0.957	1682	348	333, 231, 116
Glutamine	18.652	1.022	1785	362	347, 245, 156
Tryptophan	23.267	1.275	2251	202	377, 291, 218
Organic acids					
Pyruvic acid	7.974	0.437	1047	174	158, 131, 115
Lactic acid	8.246	0.452	1061	219	191, 147, 117
Caproic acid	8.44	0.462	1071	173	145, 131, 117
Glycolic acid	8.498	0.466	1074	205	177, 147, 133
Pyruvic acid	8.828	0.484	1091	217	189, 147, 131
Oxalic acid	9.518	0.521	1129	219	190, 147, 133
Glycolic acid	10.789	0.591	1203	147	177, 205, 233
Benzoic acid	11.615	0.636	1252	179	194, 135, 105
Phosphoric acid	12.065	0.661	1280	314	299, 283, 207
Nicotinic acid	12.44	0.681	1304	180	195, 136, 106

Succinic acid	12.579	0.689	1313	247	172, 147, 129
Glyceric acid	12.963	0.710	1339	292	220, 189, 147
Fumaric acid	13.051	0.715	1345	245	217, 147, 115
Malic acid	15.17	0.831	1496	233	189, 147, 160
Threonic acid	16.166	0.886	1574	292	220, 205, 147
Shikimic acid	19.026	1.042	1819	372	357, 204, 147
Citric acid	19.24	1.054	1839	363	347, 273, 147
Isocitric acid	19.24	1.054	1839	465	375, 273, 147
Quinic acid	19.725	1.081	1884	345	255, 204, 147
p-Coumaric acid	21.413	1.173	0	219	308, 293, 249
Sugars					
Xylose	17.402	0.953	1676	307	277, 217, 103
Galactose	19.079	1.045	1824	217	243, 204, 147
Fructose	19.936	1.092	1904	307	277, 217, 103
Fructose	20.017	1.097	1912	307	277, 217, 103
Glucose	20.2	1.107	1930	319	217, 205, 147
Mannose	20.271	1.110	1937	319	217, 205, 147
Glucose	20.413	1.118	1951	319	217, 205, 147
Glucose	20.931	1.147	2002	204	217, 191, 147
Sucrose	27.047	1.482	2705	437	361, 217, 147
Trehalose	28.068	1.538	2812	361	271, 217, 191
Sugar alcohols					
Glycerol	12.049	0.660	1279	218	205, 147, 117
Ribitol	18.255	1.000	1750	319	307, 217, 147
Mannitol	20.566	1.127	1966	319	205, 117, 103
Inositol	22.186	1.215	2132	318	305, 217, 147
Amine					
Ethanolamine	11.428	0.626	1241	174	188, 147, 100

[a] Retention time (min). [b] Relative retention times (retention time of analyte/retention time of ribitol). [c] Retention index. [d] Specific mass ion for quantification.

Figure S1. Total ion chromatogram (TIC) of hydrophilic compounds in apple mint. Peak: (1) Pyruvic acid-1; (2) Lactic acid; (3) Caproic acid; (4) Glycolic acid; (5) Pyruvic acid-2; (6) Alanine; (7) Oxalic acid; (8) Glycolic acid; (9) Valine; (10) Urea; (11) Ethanolamine; (12) Benzoic acid; (13) Glycerol; (14) Leucine; (15) Phosphoric acid; (16) Isoleucine; (17) Nicotinic acid; (18) Proline; (19) Succinic acid; (20) Glycine; (21) Glyceric acid; (22) Fumaric acid; (23) Serine; (24) Threonine; (25) β-Alanine; (26) Malic acid; (27) Aspartic acid; (28) Methionine; (29) Pyroglutamic acid; (30) 4-Aminobutyric acid; (31) Threonic acid; (32) Glutamic acid; (33) Phenylalanine; (34) Xylose; (35) Asparagine; (36) Ribitol (internal standard); (37) Glutamine; (38) Shikimic acid; (39) Citric acid; (40) Isocitric acid; (41) Galactose; (42) Quinic acid; (43) Fructose-1; (44) Fructose-2; (45) Glucose-1; (46) Mannose; (47) Glucose-2; (48) Mannitol; (49) Glucose-3; (50) p-Coumaric acid; (51) Inositol; (52) Tryptophan; (53) Sucrose; (54) Trehalose.

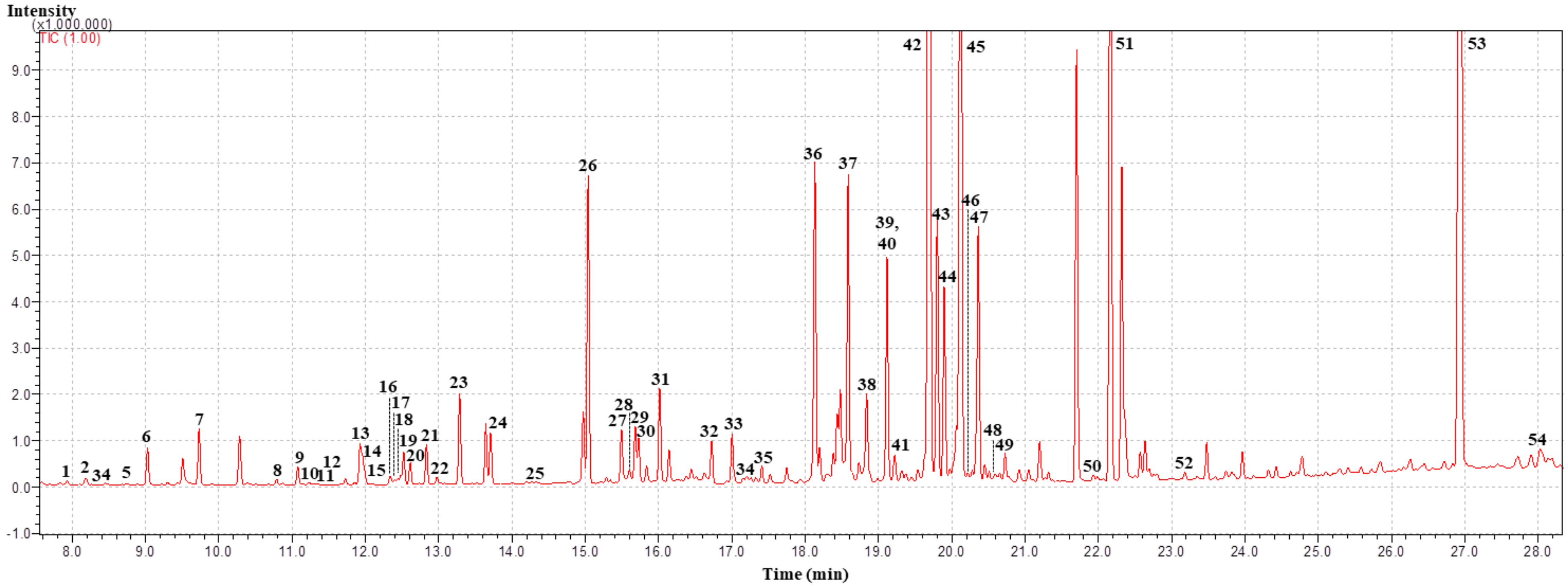

Figure S2. MS spectra of lipophilic compound standards. (1) C20, eicosanol (RT: 10.43 min); (2) C21, heneicosanol (RT: 10.98 min); (3) C22, docosanol (RT: 11.50 min); (4) C23, tricosanol (RT: 12.02 min) (5) C24, tetracosanol (RT: 12.49 min); (6) 5α-Cholestane (internal standard; RT: 13.34 min); (7) C26, hexacosanol (RT: 13.43 min); (8) β-Tocopherol (RT: 13.79 min); (9) γ-Tocopherol (RT: 13.84 min); (10) C27, heptacosanol (RT: 13.89 min); (11) C28, octacosanol (RT: 14.40 min); (12) α-Tocopherol (RT: 15.45) (13) Campesterol (RT: 15.56 min); (14) C30, triacontanol (RT: 15.59 min); (15) Stigmasterol (RT: 15.76 min); (16) β-Sitosterol (RT: 16.20 min); (17) β-Amyrin (RT: 16.47 min); (18) α-Amyrin (RT: 16.79 min).

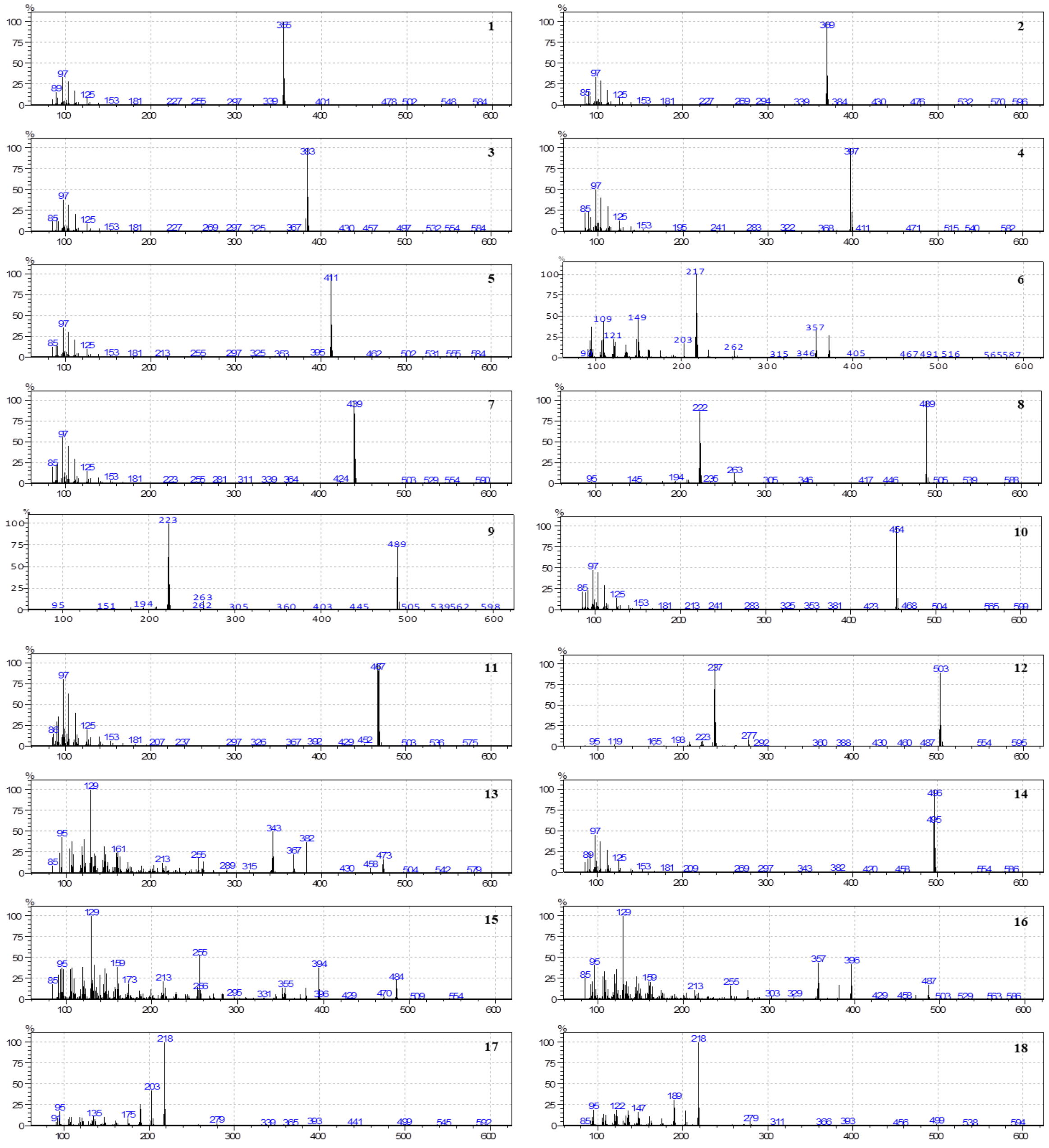

Figure S3. Extracted ion chromatogram (EIC) of lipophilic compounds in apple mint (AP). Peak: (1) C20, eicosanol (selected ion, *m/z* 355); (2) C21, heneicosanol (selected ion, *m/z* 369); (3) C22, docosanol (selected ion, *m/z* 383); (4) C23, tricosanol (selected ion, *m/z* 397); (5) C24, tetracosanol (selected ion, *m/z* 411); (6) 5α-colestane (selected ion, *m/z* 217); (7) C26, hexacosanol (selected ion, *m/z* 439); (8) β-tocopherol (selected ion, *m/z* 222); (9) γ-tocopherol (selected ion, *m/z* 223); (10) C27, heptacosanol (selected ion, *m/z* 453); (11) C28, octacosanol (selected ion, *m/z* 467); (12) α-tocopherol (selected ion, *m/z* 237); (13) campesterol (selected ion, *m/z* 343); (14) C30, triacontanol (selected ion, *m/z* 496); (15) stigmasterol (selected ion, *m/z* 394); (16) β-sitosterol (selected ion, *m/z* 357); (17) β-amyrin (selected ion, *m/z* 218); (18) α-amyrin (selected ion, *m/z* 189).

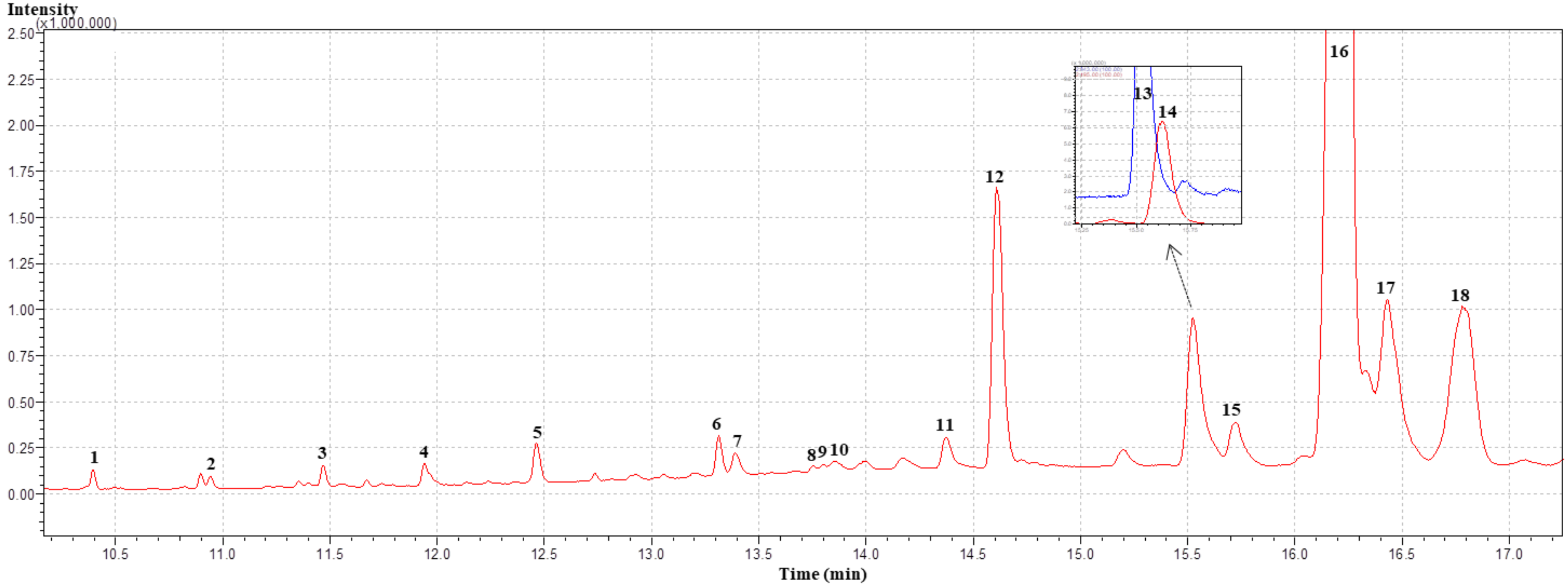

Table S2. Composition and abundance (μg/g of DW) of lipophilic compounds in nine *Mentha* species.

Compound[a]	AP	CC	ED	HS	PP	PR	PA	SP	WT
Policosanols									
C20	4.15±0.23	3.76±0.34	5.10±0.26	6.84±0.80	4.69±0.59	7.48±0.66	3.53±0.19	3.14±0.23	6.05±0.48
C21	2.04±0.10	2.07±0.37	2.15±0.16	2.32±0.16	2.10±0.25	2.16±0.31	1.99±0.13	1.95±0.48	2.25±0.14
C22	9.21±0.98	6.72±1.52	7.87±0.99	12.13±2.18	7.60±1.59	8.68±0.86	8.93±0.77	6.11±1.71	11.47±2.19
C23	2.32±0.80	1.51±0.89	1.52±0.50	3.47±1.34	2.15±1.19	1.75±0.68	2.32±0.76	1.59±1.14	2.03±0.68
C24	10.20±1.19	4.83±0.42	5.02±0.22	12.25±2.25	5.65±0.37	5.27±0.19	9.57±0.72	4.53±0.34	5.36±0.12
C26	13.72±2.59	7.60±2.02	7.26±0.66	24.18±4.60	8.55±2.00	9.46±0.74	8.92±1.44	7.09±2.46	12.61±3.33
C27	7.83±2.38	4.78±1.30	5.77±0.07	11.95±3.43	5.92±1.27	6.00±0.64	6.03±0.52	5.70±1.34	6.30±1.41
C28	19.57±2.86	23.56±7.75	16.00±2.62	28.49±7.29	15.96±3.04	17.82±1.07	13.49±2.00	16.57±4.52	33.37±13.34
C30	57.22±19.00	48.89±21.70	31.01±8.40	19.94±6.75	23.78±8.34	13.21±2.40	43.56±9.83	12.04±4.10	90.54±43.19
Total	126.26±30.13	103.72±36.31	81.70±13.87	121.59±28.80	76.40±18.64	71.82±7.55	98.34±16.36	58.73±16.33	169.97±64.88
Amyrins									
α-Amyrin	61.70±6.64	121.72±22.04	270.71±26.96	114.40±14.97	84.30±13.53	161.14±13.60	36.74±3.02	68.82±12.12	272.51±31.14
β-Amyrin	54.78±5.69	43.81±5.36	97.45±11.60	126.20±20.22	101.36±19.72	44.18±2.36	35.75±3.44	72.50±14.45	130.17±11.59
Sterols									
Campesterol	188.55±22.70	177.24±40.67	129.76±19.57	149.47±29.88	112.99±22.95	372.87±42.25	181.26±19.12	141.32±24.13	151.64±32.79
β-Sitosterol	986.15±111.51	635.26±116.46	585.25±69.39	822.95±126.35	688.17±136.82	807.27±86.90	826.78±126.04	741.01±162.58	799.67±125.61
Stigmasterol	59.37±9.16	78.48±22.03	102.88±12.69	51.14±9.82	72.80±14.69	34.73±2.90	70.75±14.79	72.58±18.87	116.49±21.73

Tocopherols									
α-Tocopherol	96.02±9.37	71.79±7.52	114.04±8.86	141.58±11.86	119.23±19.50	69.93±3.29	82.10±7.15	76.32±11.89	101.79±13.66
β-Tocopherol	2.14±0.06	2.01±0.06	2.24±0.07	1.96±0.01	2.18±0.24	1.89±0.07	2.13±0.11	1.97±0.13	1.97±0.12
γ-Tocopherol	2.41±0.20	2.09±0.28	2.13±0.03	2.33±0.12	3.25±0.16	2.78±0.13	2.60±0.11	2.86±0.28	2.27±0.11
Total	1451.12±165.34	1132.40±214.43	1304.47±149.19	1410.03±213.23	1184.29±227.62	1494.80±151.51	1238.11±173.76	1177.38±244.45	1576.51±236.74

[a] C20, eicosanol; C21, heneicosanol; C22, docosanol; C23, tricosanol; C24, tetracosanol; C26, hexacosanol; C27, heptacosanol; C28, octacosanol; C30, triacontanol. Each value is the mean of three replications ± standard deviation. AP, apple mint; CC, chocolate mint; ED, eau de cologne mint; HS, horse mint; PP, peppermint; PR, pennyroyal mint; PA, pineapple mint; SP, spearmint; WT, water mint.

Figure S4. : LC chromatogram of phenolic compounds in *Mentha* species. (A) Apple mint; (B) Chocolate mint; (C) Eau de cologne mint; (D) Horse mint; (E) Peppermint; (F) Pennyroyal mint; (G) Pineapple mint; (H) Spearmint; (I) Water mint.

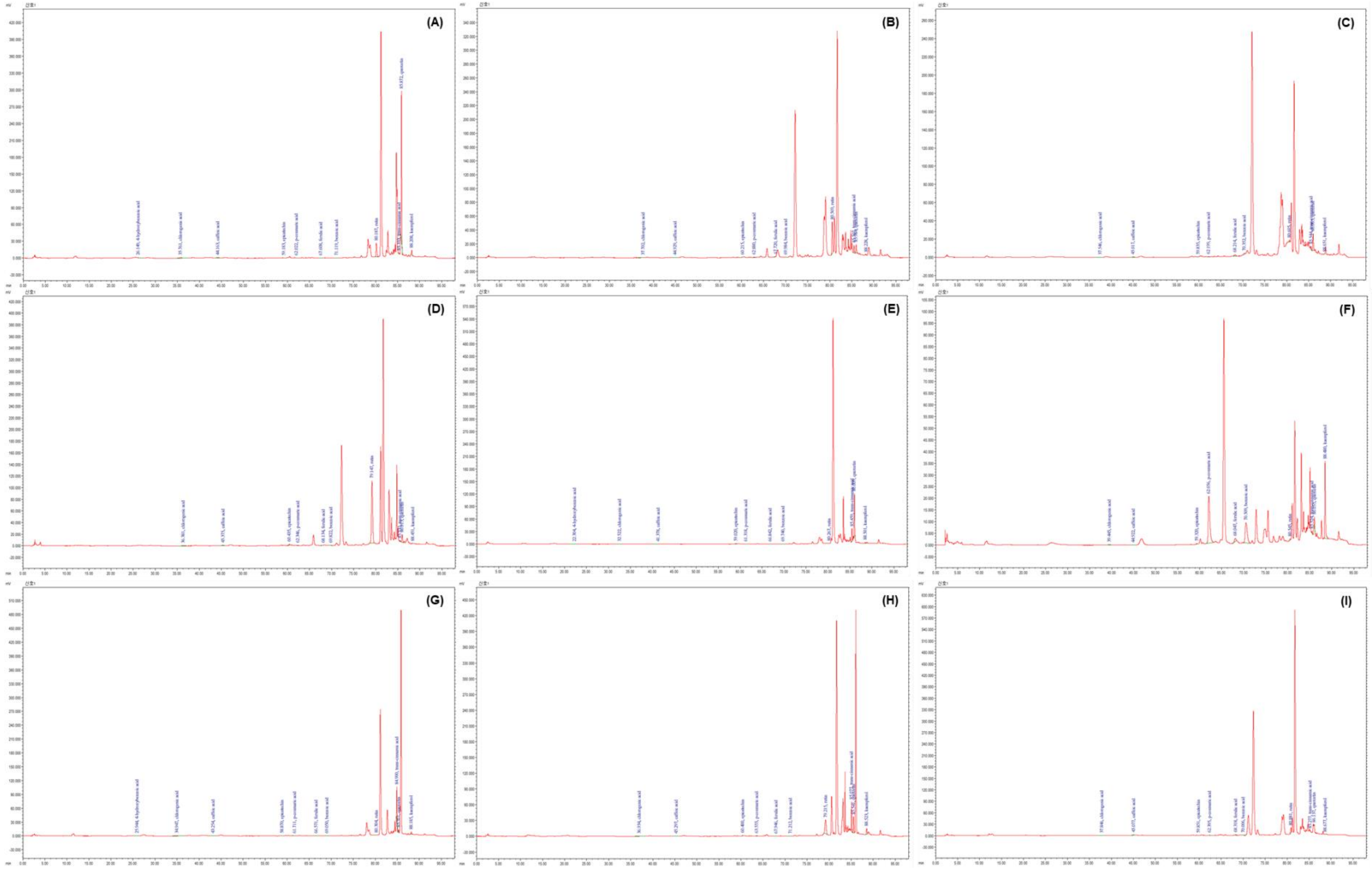

Figure S5. LC chromatogram of riboflavin in *Mentha* species. (A) Apple mint; (B) Chocolate mint; (C) Eau de cologne mint; (D) Horse mint; (E) Peppermint; (F) Pennyroyal mint; (G) Pineapple mint; (H) Spearmint; (I) Water mint. The Fluorescence excitation at 430 nm; fluorescence emission at 540 nm.

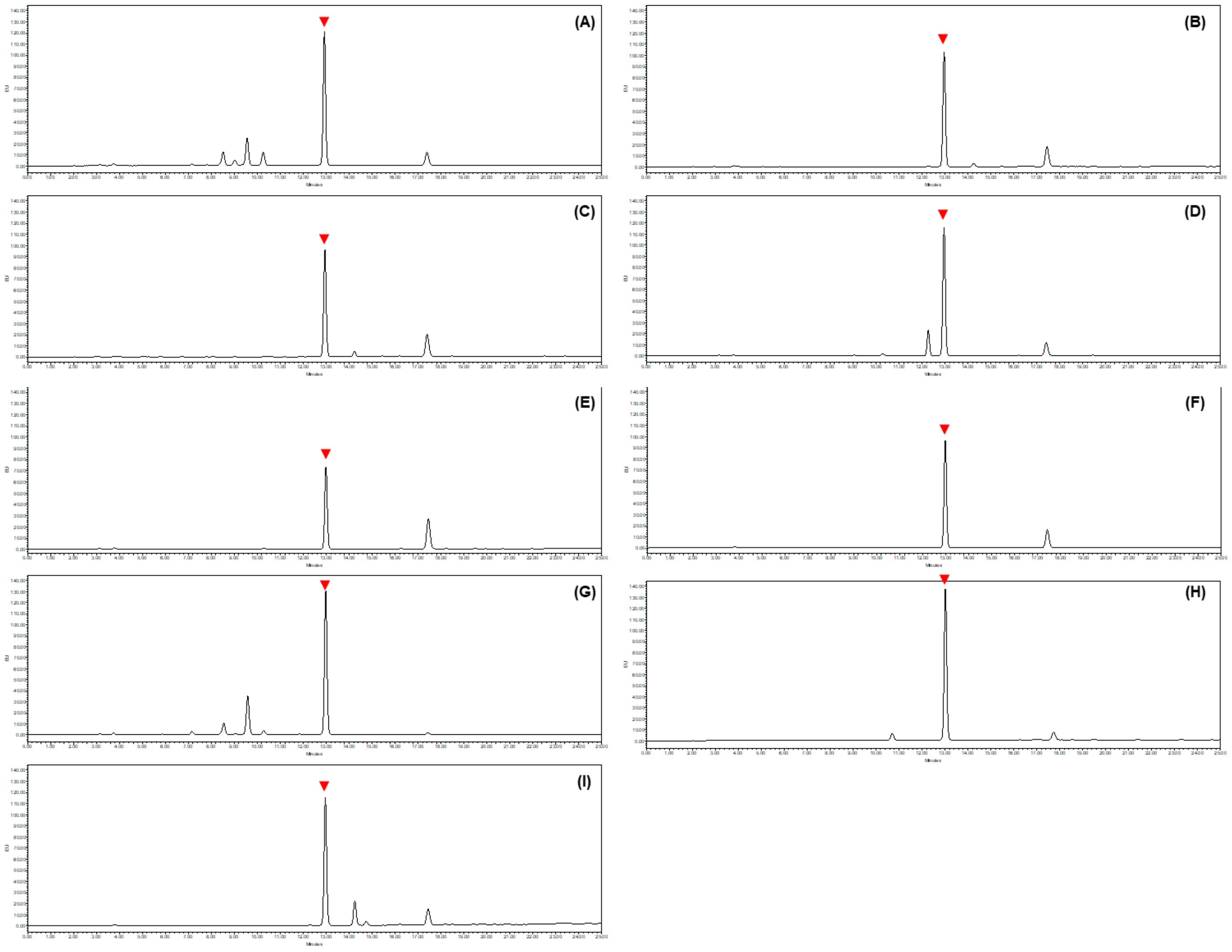

Figure S6. LC chromatogram of carotenoids in *Mentha* species. (A) Apple mint; (B) Chocolate mint; (C) Eau de cologne mint; (D) Horse mint; (E) Peppermint; (F) Pennyroyal mint; (G) Pineapple mint; (H) Spearmint; (I) Water mint. Peak: (1) Violaxanthin; (2) Antheraxanthin; (3) Lutein; (4) Zeaxanthin; (5) trans-β-Apo-8'-carotenal (internal standard); (6) 13Z-β-carotene; (7) α-Carotene ; (8) E-β-Carotene; (9) 9Z-β-Carotene.

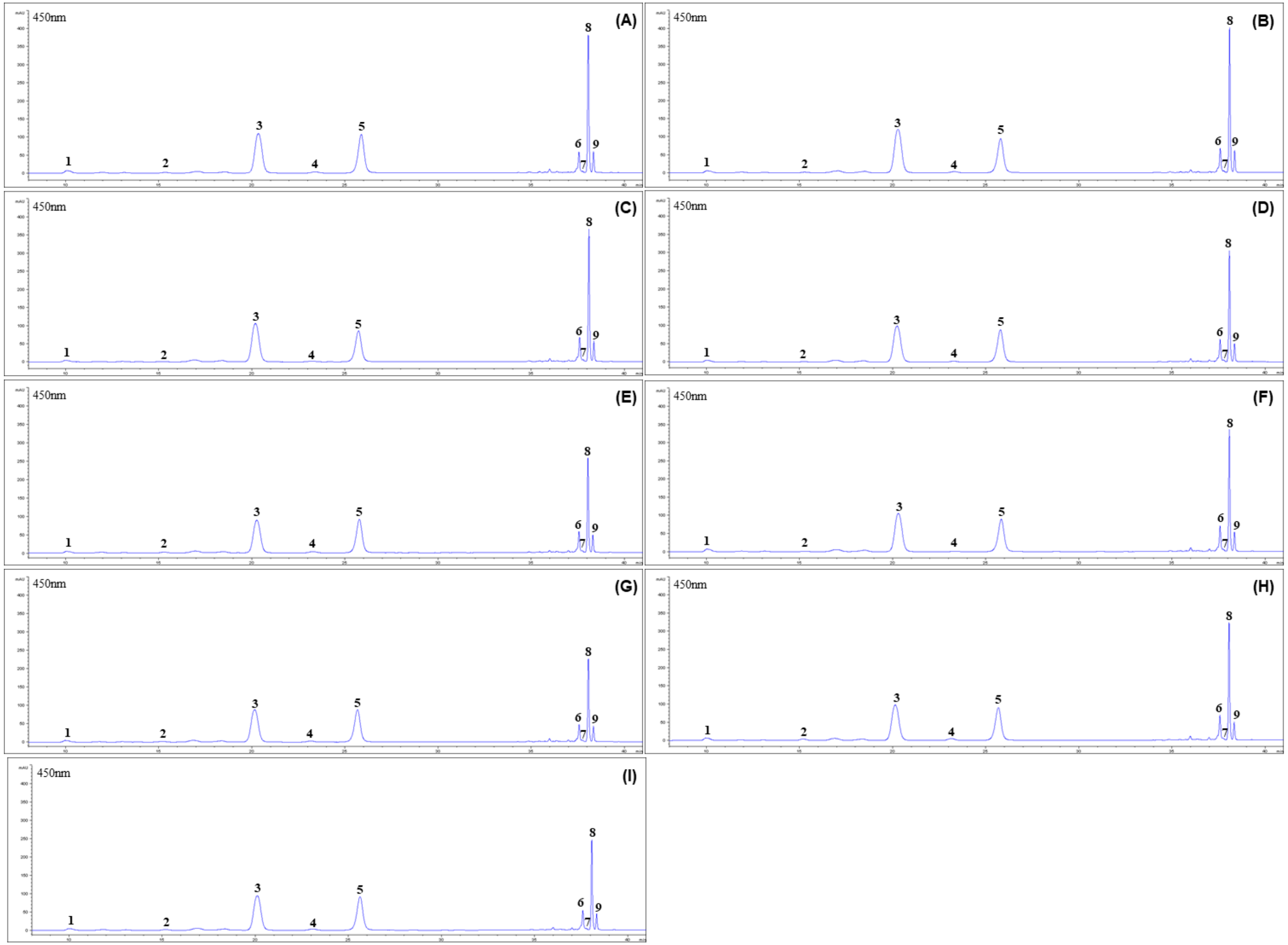

Figure S7``. Relationship between total carotenoid content and reducing power assay of *Mentha* species.

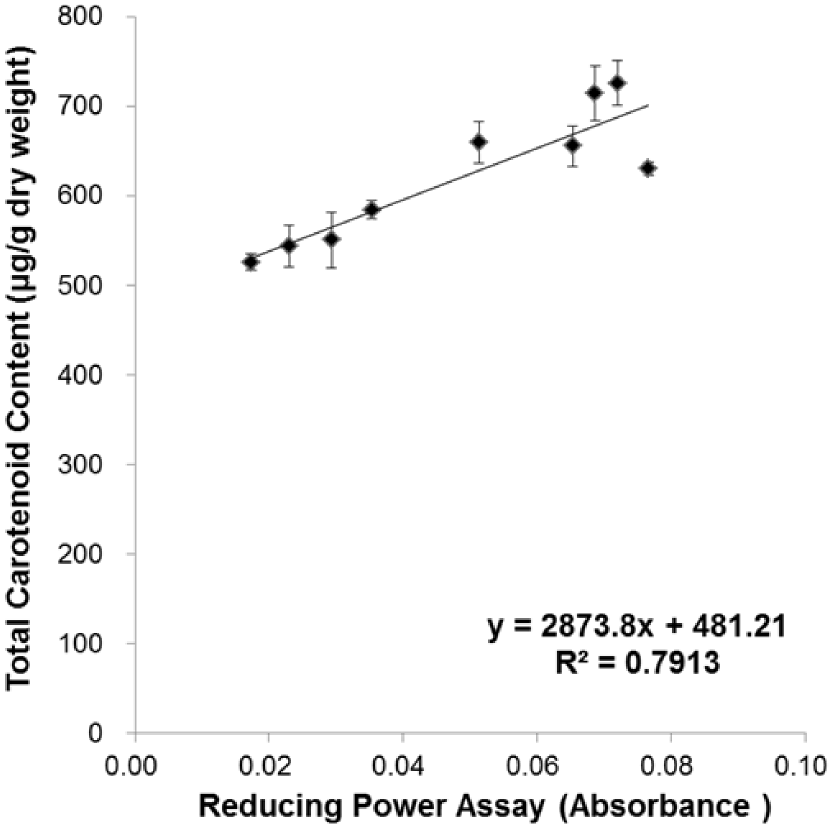

Supplement: Supplementary file 1 [file molecules-24-00258-s001.pdf]
